# An inter-laboratory trial as a tool to increase rabies diagnostic capabilities of Sub-Saharan African Veterinary laboratories

**DOI:** 10.1371/journal.pntd.0008010

**Published:** 2020-02-10

**Authors:** Morgane Gourlaouen, Angélique Angot, Marzia Mancin, Charles Bebay, Baba Soumaré, Francesca Ellero, Barbara Zecchin, Stefania Leopardi, Cristian De Battisti, Calogero Terregino, Paola De Benedictis

**Affiliations:** 1 FAO and National Reference Centre for Rabies, OIE Collaborating Centre for Diseases at the Animal-Human Interface, Istituto Zooprofilattico Sperimentale delle Venezie, Legnaro (PD), Italy; 2 Laboratory Unit—Emergency Prevention System (EMPRES), Animal Health Service, Food and Agriculture Organization of the United Nations (FAO-UN), Rome, Italy; 3 Risk Analysis and Public Health Department, Istituto Zooprofilattico Sperimentale delle Venezie, Legnaro (PD), Italy; 4 Food and Agriculture Organization of the United Nations (FAO), Emergency Center for Transboundary Animal Diseases (ECTAD), Nairobi, Kenya; 5 Food and Agriculture Organization of the United Nations (FAO-UN), Emergency Center for Transboundary Animal Diseases (ECTAD), Accra, Ghana; US Department of Agriculture, UNITED STATES

## Abstract

To achieve the goal of eliminating dog-mediated human rabies deaths by 2030, many African countries have agreed to list rabies as a priority zoonotic disease and to undertake both short and long-term control programs. Within this context, reliable local diagnosis is essential for the success of field surveillance systems. However, a harmonized, sustainable and supportive diagnostic offer has yet to be achieved in the continent. We herewith describe the organization and outcome of a proficiency test (PT) for the post-mortem diagnosis of rabies in animals, involving thirteen veterinary laboratories and one public health laboratory in Africa. Participants were invited to assess both the performance of the Direct Fluorescent Antibody (DFA) test and of a conventional RT-PCR. From the submitted results, while thirteen laboratories proved to be able to test the samples through DFA test, eleven performed the RT-PCR method; ten applied both techniques. Of note, the number of laboratories able to apply rabies RT-PCR had increased from four to ten after the exercise. Importantly, results showed a higher proficiency in applying the molecular test compared to the DFA test (concordance, sensitivity and specificity: 98.2%, 96.97% and 100% for RT-PCR; 87.69%, 89.23% and 86.15% for DFA test), indicating the feasibility of molecular methods to diagnose animal pathogens in Africa. Another positive outcome of this approach was that negative and positive controls were made available for further in-house validation of new techniques; in addition, a detailed questionnaire was provided to collect useful and relevant information on the diagnostic procedures and biosafety measures applied at laboratory level.

## Author summary

Although Africa has the highest per capita death rate from rabies, the incidence of canine rabies on the continent remains a matter that needs to be further investigated; in addition, the lack of accurate information impairs the establishment of long-term actions to progress towards a rabies free status. In this scenario, creating efficient diagnostic facilities is of utmost importance for eradicating the disease and implementing effective surveillance programs in endemic areas. With the final objective of eliminating dog-mediated human rabies globally by 2030, sample submission to African veterinary laboratories for rabies diagnostic testing is expected to increase. Furthermore, in compliance with ISO/IEC 17025:2017 standard, participating to proficiency testing (PT) exercises becomes mandatory not only to regularly assess the validity of the in-house protocols but also to harmonize the techniques. Thirteen veterinary laboratories and one public health laboratory in Africa were invited to take part in the exercise in response to an invitation from the Istituto Zooprofilattico Sperimentale delle Venezie (IZSVe), Italy, which hosts the FAO rabies Reference Center. This study presents the results obtained by applying the DFA test and the conventional RT-PCR method to the PT panel, which show that even if laboratories preferably apply the DFA test to detect the presence of rabies in animal samples, the overall concordance of results was higher through molecular testing. Importantly, the organization of the exercise proved to be a good opportunity to update the vaccine cover status of laboratory staff.

## Introduction

Rabies virus still claims more than 59,000 human lives every year and affects lower socio-economic groups in resource-poor countries. In Africa, where the highest per-capita death rate is accounted for [1], rabies has been listed as a priority zoonotic disease through the national zoonotic disease prioritization process using the Centre for Disease Control (CDC) One Health Zoonotic Disease Prioritization Tool (OHZDPT) in many African countries [2]. The tripartite collaboration of World Health Organization (WHO), World Organisation for Animal Health (OIE) and Food and Agriculture Organization of the United Nations (FAO), together with the non-profit organisation Global Alliance for Rabies Control (GARC) have set up the goal to end dog-mediated human rabies by 2030. The elimination of canine rabies in Africa is achievable through the firm political will and engagement of local stakeholders, who are required to take on responsibilities and perform actions for the cause [3]. Tools are available to support and guide countries in developing national programs and strategies for sustainable rabies prevention, rabies control and gradually progress towards rabies elimination [4]. In this context, estimating the disease burden relies on accurate surveillance plans in the field. Performant diagnostic facilities require the availability of well-maintained infrastructures and experienced laboratory technicians to perform the recommended protocols. An accurate diagnosis of animal rabies cases is crucial not only to monitor the progress of control efforts but also to advise in the clinical management of patients that have been potentially exposed to the virus.

The recent revision of the OIE Manual of Diagnostic Tests and Vaccines for Terrestrial Animals includes validated RT-PCR methods and two antigen-based techniques, the direct immunochemical test (dRIT) jointly with the Direct Fluorescent Antibody test (DFA test) as gold standard for post-mortem diagnosis of animal rabies. The DFA was previously the only gold standard technique available to perform post mortem animal diagnosis from brain tissues [5]. Although it is a simple, rapid and cheap method that is also highly sensitive and specific on fresh specimens [6], the DFA test is currently under-exploited in low-resource areas. The causes for such a partial success are in part due to the technique itself, but also to the limited accessibility of central laboratories from remote areas to the reagents needed to perform the DFA test. If on one hand they are cheaper than those used for conventional RT-PCR testing, their limited shelf life upon reconstitution that requires a regular supply of specific material such as fluorophore labelled anti-rabies antibodies, as well as the costs of acquiring and maintaining a fluorescent microscope, are believed to be the main constraints for this technique [7]. In addition, the possible misinterpretation of the test by inexperienced staff should not be underestimated [4]. Among the alternative techniques now recommended by the OIE, RT-PCR certainly overcomes some of these issues. Indeed, molecular techniques do not require any personal interpretation, meaning that misinterpretation errors are reduced to a minimum and can be more easily utilize in veterinary laboratories across Africa, which have become increasingly more equipped and better trained to perform rapid diagnosis of Avian Influenza and other transboundary diseases [8][9]. Furthermore, molecular techniques target the viral genome rather than the antigen, likely increasing sensitivity and specificity in samples with a bad preservation status compared to the DFA test, where the antigen might be degraded during putrescence and/or secondary bacterial infection may lead to unspecific fluorescence [10]. In any case, the selected conventional protocol constitutes a major diagnostic tool thanks to its ability to rapidly detect all known lyssaviruses in both animal and human samples [11]. Moreover, the product of amplification allows for identification of the virus involved in the infection through sequencing [11] [12].

We herewith describe the organization and outputs of a PT exercise specifically designed to assess the performances of fourteen laboratories across West, Central and East Africa in post-mortem diagnosis of animal rabies. Participants were asked to apply the DFA test, which at the time of the PT organization was the only recommended gold standard test, along with a broad spectrum one step-RT PCR [11]. Both techniques allow the detection of different viral targets, which is an important issue considering the high variability of the material that is submitted to the laboratories (type of sample, level of preservation, putrefied samples, inactivated smear on FTA cards). Ultimately, the PT exercise offered the opportunity to validate the implementation of a molecular test for the post-mortem diagnosis of rabies in most participating laboratories, and made it possible to collect information related to laboratory practices and vaccination status of laboratory members. Although previous studies have described such an exercise for European and North African [13], Latin American and the Caribbean laboratories [14], to our knowledge this is the first study describing the results of a PT for Sub-Saharan African laboratories.

## Materials and methods

### Participating laboratories

Thirteen National Central Veterinary Laboratories (CVLs) and one Public Health Institute were included in the PT program, all were based in Global Health Safety Agenda (GHSA)/Emerging Pandemic threats (EPT-2) beneficiary countries, i.e. Burkina Faso (Laboratoire National d’Elevage (LNE) de Ouagadougou), Cameroon (Laboratoire National Vétérinaire (LANAVET) Garoua and LANAVET annexe Yaoundé), Côte d’Ivoire (Laboratoire National D’Appui au Développement Agricole (LANADA)), Democratic Republic of Congo (Laboratoire Vétérinaire de Kinshasa), Ghana (Accra Veterinary Laboratory), Guinea (Laboratoire Central de Diagnostic Vétérinaire de Conakry), Mali (Laboratoire Central Vétérinaire (LCV) du Mali), Senegal (Laboratoire National d’Elevage et de Recherches Vétérinaires (LNERV)), Ethiopia (Ethiopian Public Health Institute (EHPI)), Kenya (Central Veterinary laboratories), Liberia (Leon Quist Ledlum Central Veterinary diagnostic Laboratory), Tanzania (Tanzania Veterinary Laboratory Agency (TVLA)) and Uganda (National Animal Disease Diagnostic and Epidemiology Centre (NADDEC)). All thirteen veterinary laboratories operate under the Ministries of Agriculture in their countries, whilst the public health institute operates in the framework of the Ministry of Health.

### Panel composition

The panels included 12 freeze-dried samples (10 unknown coded samples plus a positive and a negative control) (Table 1). Each sample contained 1 ml of brain material to be resuspended with 1 ml of sterile distilled water.

**Table 1:** Panel composition and expected classification of samples. Panels included ten freeze-dried blinded samples, one positive and one negative control. Five out of ten samples were expected to turn out positive through DFA test, whilst six out of ten samples in the panel were expected to be positive using the RT-PCR test. RABV (Rabies virus) and DUVV (Duvenhage virus), all belonging to phylogroup I, were selected for the exercise.

Sample ID	Virus / Lineage	Expected classification DFA test/RT-PCR test	Infected material concentration (%)	Fluorescence intensity
**1**	FIXED RABIES STRAIN/CVS-11	+/+	10	3+
**2**	RABV DOG / Africa 2	+/+	12.5	3+
**3**	RABV DOG / Cosmopolitan (exAfrica 1)	+/+	15	4+
**4**	DUVV (South Africa 1971)	+/+	20	2+
**5**	RABV HONEY BADGER / Africa 3	+/+	15	4+
**6**	Uninfected CNS	-/-	/	0
**7**	Uninfected CNS	-/-	/	0
**8**	Uninfected CNS	-/-	/	0
**9**	Uninfected CNS containing lipofuscin	-/-	/	Non-specific staining
**10**	Uninfected CNS containing lipofuscin and synthetic RNA	-/+	*	Non-specific staining
**Positive Control**	FIXED RABIES STRAIN/CVS-11	+/+	40	4+
**Negative Control**	Uninfected CNS	-/-	/	0

* The final concentration of synthetic RNA is 750 ng/ml. CNS (Central Nervous System).

Positive samples consisted of a mixture of homogenised central nervous system (CNS) from infected mice and CNS from uninfected mammals. Each batch of virus was prepared by intra-cerebral inoculation of three weeks old CD1 mice following a refined anaesthetic protocol [15][16]. Animals were observed twice a day and were humanely sacrificed when the severity of symptoms impaired access to food and water [5].

Uninfected material consisted of brain tissue collected from wild and domestic mammals under the framework of passive surveillance (fox, dog and cat), which tested negative for rabies by means of DFA test, Rabies Tissue Culture Infection Test (RTCIT) [5] and RT-PCR [11]. Possible infection of those samples with canine distemper virus was also ruled out by means of RT-PCR [17]. For the preparation of two out of the five negative samples present in each panel, negative CNS material containing lipofuscin granules was specifically selected to mimic common findings in field brain tissues. Lipofuscin or autofluorescent lipopigment naturally accumulates within the CNS tissues of aging animals [18]. Concretely, when the DFA test slides are analyzed under the fluorescent microscope, lipofuscin appears as a ubiquitous unspecific golden staining and this observation occurs regardless of specific antigen-antibody complexes [19]. Furthermore, one of the rabies uninfected samples was spiked with an RNA synthetically produced at the IZSVe and contained the N gene sequence of rabies virus (Challenge Virus Strain -11). This particular sample was expected to be negative using the DFA test, which targets the viral antigen but should be positive by molecular testing, which in fact targets the viral RNA. In summary, five samples out of ten composing the panel were expected to be positive through DFA test, whilst six out of ten samples were expected to be positive using the RT-PCR protocol (Table 1).

The panel included strains with epidemiological relevance in the area, field Rabies viruses (RABV) representing 3 lineages circulating in Africa, i.e. Cosmopolitan ex-Africa 1 (EX-AFR1), Africa 2 (AFR2), Africa 3 (AFR3) and a Duvenhage virus (DUVV) representing an African bat-associated virus, all belonging to phylogroup I. Divergent lyssaviruses (LYSVs) were intentionally discarded for safety issues, despite circulating in flying and non-flying wildlife in the African continent. As a matter of fact, the protection developed from currently available vaccines against rabies related lyssaviruses belonging to phylogroups II and putative III/IV is still debated [20].

To avoid any possible contamination, samples were prepared in different batches on different days, with particular attention during the addition of synthetic RNA, which was performed once all other samples were already prepared. Lyophilisation of samples was achieved following adapted freeze-dry protocol in a lyophilisator minifast 6000 (Edwards) and samples were kept at -20°C. Adapted decontamination of lyophilisator was performed between each batches by manual cleaning using 1% Virkon solution followed by a high heat decontamination cycle.

#### Ethical statement

This study included (i) the use of laboratory animals and (ii) the use of samples collected and submitted to the laboratory and tested within the diagnostic activities carried out at the FAO Reference Centre (RC) for rabies.

(i) All experimental procedures involving laboratory animals were performed in strict accordance with the relevant national and local animal welfare bodies [Convention of the European Council no. 123 and National guidelines (Legislative Decree 26/2014)]. The protocol was approved by the Committee on the Ethics of Animal Experiments of the IZSVe (CE.IZSVe20/2014) and then authorized by the Italian Ministry of Health (Decree 505/2015-PR) before experiments were initiated.

(ii) The brain tissue obtained from archived CNS samples of non-endangered mammals was collected in the framework of the national passive surveillance for rabies. All animals were found dead or legally euthanized by a competent veterinarian. According to the national legislation regulating animal experimentation, no ethical approval or permit was required for collecting and processing this type of specimen. All samples used for the PT were also tested for canine distemper virus to avoid unintentional spread of an exotic strain of relevance for both conservation and animal health.

### Synthetic RNA preparation

*E*.*coli* colonies transfected with a plasmid pCR2.1 (Invitrogen) containing the N gene sequence from rabies virus (strain CVS-11) were retrieved from the archive (full sequence available upon request). Colonies were grown overnight at 37°C and plasmids were extracted using a commercial kit (genElute, Sigma-Aldrich (Merck)). The region of interest was amplified by conventional PCR (AmpliTaq Gold DNA Polymerase, Thermo Fisher) using M13 forward (5´-GTAAAACGACGGCCAG-3´) and reverse (5´-CAGGAAACAGCTATGAC-3´) primers. The product of amplification was then applied to an agarose gel and the band was extracted using the QIAquick Gel Extraction Kit (Qiagen). Transcription was performed using MEGAscript T7 transcription kit (Thermo Fisher Scientific) and synthetic RNA was then extracted using MEGAclear kit (Thermo Fisher Scientific). The quantity of RNA was measured by nanodrop (Thermo Fisher) and stored at -80°C until use. Synthetic RNA at a final concentration of 750 ng/ml and RNase inhibitor (Promega) were then added to the brain mixture at the final concentration of 1IU/μl before distributing the material among the vials. Homogeneity and stability were confirmed by RT-PCR [11]. Sanger sequencing was applied during the homogeneity control check to confirm sample quality and to exclude the occurrence of any possible contamination with other samples.

### Quality control tests

Several quality control tests were performed on samples during the whole preparation process. As recommended by the ISO 17043, for fewer than 100 specimens [21], homogeneity was controlled post lyophilisation by testing a minimum of 10% of vials in duplicate by DFA test and RT-PCR test. Samples collected prior the freeze dry process were also tested in parallel to assess the effect of lyophilisation. Several controls of sample stability were conducted during the whole testing period, which included testing two panels which were exposed to 37°C for 14 days to mimic improper transport and/or unsuited storage conditions and two panels stored at -20°C for 6 weeks. Effect of long term storage was also assessed by testing panels kept at -20°C for 6 and 12 months (after the reception of the last PT results). Panels were tested using both techniques requested for the PT. Following rehydration of the vials, slides for the DFA test were prepared by smear technique [5]. Slides were fixed overnight using 100% acetone at -20°C, air-dried for 15 minutes and stained using a commercial anti-rabies nucleocapsid conjugate (Bio-Rad, 3572112). CNS was diluted 1:10 in Phosphate Buffer Saline (PBS) and One-step RT-PCR was performed as described [11]. Prior the lyophilisation step and during the homogeneity control, products of RT-PCR were further characterised by Sanger sequencing to confirm the presence of the specific strains and exclude any cross contamination between PT samples. Quality and stability outcomes for all the tested vials corresponded to the expected results, as indicated in Table 1.

### General organization

Laboratory Directors from twelve laboratories were formally invited to participate to the PT rabies 2017 by an official e-mail sent on 28^th^ September 2017. This date celebrates the World Rabies Day and was purposely picked to sensitize all participating laboratories. The invitation of the remaining two laboratories was postponed to October and November 2018 due to a lack of functioning laboratory facilities. FAO Teams and FAO ECTAD Regional Laboratory experts in Western and Central Africa were included in all email communication throughout the PT exercise. A rabies vaccination status datasheet was also sent to each laboratory alongside the invitation letters. Laboratories not presenting a correct immunization coverage of the staff were informed that shipment of their panel would be postponed until completion of the vaccination process. The following additional documents in English or in French were also provided: i) instructions for rabies samples handling, resuspension and storage (S1 Appendix) ii) protocols of the DFA test and the recommended conventional RT-PCR [11] (S2 Appendix).

In order to set favorable and safe conditions for the participating laboratories, a webinar (English version http://fao.adobeconnect.com/pxusvkh7qf6f/ and French version http://fao.adobeconnect.com/pgk7jufqhhdf/) was co-organized with FAO laboratory Unit (HQ) three weeks before shipping the panels. The aim of this webinar was to consolidate knowledge about Biosafety and Biosecurity procedures for handling suspected rabies samples and to offer a tutoring on how to perform a successful PT exercise.

Amongst the activities coordinated by FAO, laboratories also received support for the maintenance of infrastructure and equipment of facilities, as well as specific reagents under request. Although a protocol for DFA was informatively distributed, laboratories were invited to test the PT panel by means of the methodology routinely applied using the reagents and equipment available at their respective diagnostic facilities. Of note, laboratories already using RT-PCR for the diagnostic of rabies before this PT applied the protocol recommended by the FAO RC [11]. The laboratories which did not apply RT-PCR to diagnose rabies were invited to use the FAO RC recommended protocol [11]. Thus, given the portion of participants which did not possess the recommended primers, they were provided to all participants. A few days before the shipment, laboratories received via e-mail a questionnaire related to the PT and the quality system in place (S3 Appendix) together with a result form. Aim of the questionnaire was to assess good laboratory practices (GLPs), biosafety and biosecurity (BB) procedures, available resources to eventually evaluate the variability of the diagnostic methods, applied protocols and workload.

### Panel shipment

Panels were sent on dry ice (-80°C) under UN2814 and UN1845 conditions, in strict compliance with the international regulations on transport of dangerous goods (WHO, 2015), through a dedicated courier company. In order to control the quality of shipment, TimeStrip Plus (Sigma) was placed inside each parcel to record whether the panels were exposed to a temperature above 10°C for up to 48 hours. In addition, all laboratories were asked to report on the condition of the parcel at arrival, in order to consider possible deterioration of material due to inappropriate storage in case of failure of results.

### Proficiency testing

Upon reconstitution of the lyophilized materials, the participants were invited to test the coded vials using both techniques. Importantly, participants were asked to score samples as positive or negative, but qualitative assessment of the fluorescence observed by DFA test was not expected. Participants were requested to submit their results within four weeks of receiving the panel. Although participants presenting discordant results were invited to re-test PT samples, the results included in this study were the ones submitted after the first attempt.

### Data and statistical analysis

Statistical analysis of results was performed in agreement with ISO 13528 [22], which suggests assessing laboratory performances in proportion to the number of correct results obtained from qualitative testing. Sensitivity, specificity, intra and inter laboratory concordance and Cohen’s kappa coefficient (K) were calculated for both techniques (DFA and RT-PCR). Sensitivity and Specificity respectively measure the percentage of positive and negative samples identified correctly. The intra- and inter-laboratory concordance correspond to the percentage of answers in agreement with the expected results for each laboratory and for each sample, respectively. Finally, the K statistic evaluates the agreement of the results for each laboratory taking into account the agreement occurring by chance. K value can be interpreted using Landis and Koch’s evaluation scale [23]. The acceptance criteria considered as a positive outcome the performance of laboratories that had reached a substantial agreement (when a minimum K of 0.61 was obtained). However, due to the relevance in terms of public health in failing to identify a case of rabies, the outcome of the PT was considered unfavourable when laboratories failed to identify one or more rabies positive samples, regardless of the agreement value obtained. The performance of each test included in the PT (DFA, RT-PCR) was graphically evaluated using a scatterplot of sensitivity by specificity for each laboratory. For a general evaluation of the PT, overall sensibility, specificity, concordance and Cohen’s K statistic among the laboratories were also calculated.

Moreover, a weighted K coefficient was computed to include the maintenance of technical skills based on the number of routine diagnostic analysis, as reported in the provided questionnaire. More in detail, the weight of an error in rabies diagnosis was classified as follows: “light” for laboratories analysing less than 5 samples a month, “light-medium” for laboratories analysing between 5 and 15 samples a month, and “medium” for laboratories analysing more than 15 samples. Furthermore, in agreement with the acceptance criteria, the weight of a false negative result was higher than the one obtained from a false positive.

Furthermore, statistical analysis was also performed to highlight relevant factors (e.g. fixation method, type of fluorescence conjugate, number of samples routinely analysed, respecting the results submission deadline) that might have affected the results (in agreement/disagreement). Fisher’s exact test or Pearson's Chi-squared test (according to the expected frequencies of the contingency table cells) were conducted. To quantify the magnitude of the evaluated association, the Cramer’s V was calculated [24]. P-values <0.05 were considered significant. STATA 12.1 software [25] and R version 3.4.1 [26] were used to conduct the statistical analysis.

## Results

Fourteen laboratories operating within the framework of either the Ministry of Agriculture or the Ministry of Health in thirteen African countries were invited to participate in the PT exercise and they all accepted. Laboratory directors were required to ensure that all laboratory staff likely to be in contact with rabies suspected samples were immunized against rabies as recommended by the WHO guidelines [27]. However, five out of the fourteen (5/14, 35.7%) participating laboratories did not present satisfactory vaccination coverage. In order to receive their PT panel, laboratories were invited to undertake either the entire pre-exposure prophylaxis (PrEP) or a booster dose according to the staff vaccination history. Members from eight laboratories attended the pre-PT webinar; however, all PT participants were sent a link to re-view the session also at a later time, if needed.

**Fig 1:**
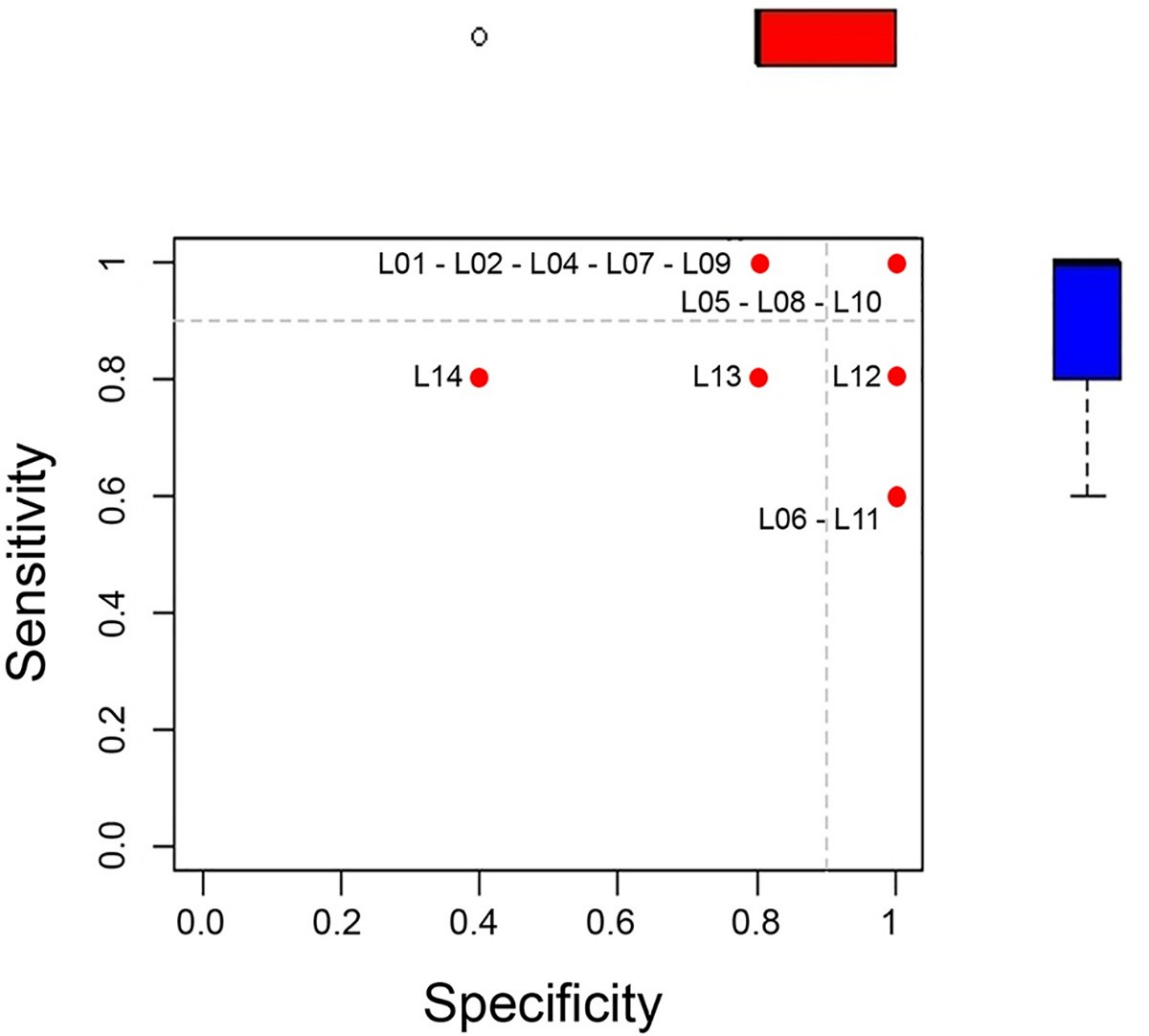
Scatter plot of sensitivity by specificity of DFA PT results. The sensitivity (y-axis) and the specificity (x-axis) are shown for each laboratory (red dot and laboratory ID). Laboratories 05, 08 and 10 correctly identified all samples and can be found in the upper right corner. The box-plots displayed in the margin represent the data distribution of the sensitivity (blue) and specificity (red).

All CVLs reported the delivery of their panels in good condition. The average length of parcel delivery to the recipient laboratory was 11 days, ranging from 4 days to a maximum of 56 days. Length in shipment did not affect the results (pχ^2^ = 0.84), which were submitted by all laboratories. In particular, eleven out of fourteen laboratories (78.6%) respected the four-week deadline for results submission, whilst three laboratories (21.4%) provided results 34 days, 80 days and nearly 11 months (308 days) after receiving the PT panels (Fig 1). To exclude that degradation in the quality of the PT samples may account for this result, whole PT panels were tested after 6 and 12 months by the PT provider and all results were as expected. Overall, ten out of fourteen laboratories (71.4%, 10/14) were able to analyse the coded samples using both techniques. Two laboratories (20%, 2/10) correctly identified all samples using both the DFA test and the RT-PCR techniques (100% sensitivity and specificity) (Table 2 and Table 3). Whilst sixteen discordant results were found by DFA test; i.e. nine false positive (FP) and seven false negative (FN), only two FN were found by RT-PCR. Interestingly, a delay in the submission of results was associated with a greater inaccuracy in detection (pχ^2^ = 0.017); the presence of discordant results was higher in laboratories with difficulties in respecting the deadline.

**Table 2:** Fluorescent antibody test results by laboratory and statistical analyses of agreement. The concordance, specificity, sensitivity and K coefficient with p-value were calculated per each laboratory. Thirteen laboratories (out of fourteen) submitted results for this part of the PT. Laboratory L03 did not submit any results by DFA test. Three laboratories (L05, L08 and L10) presented a full agreement (K = 1) and 100% concordance with the results expected by the PT organiser.

Laboratory	True positive	True negative	False positive	False negative	Concordance (%)	Sensitivity (%)	Specificity (%)	Kappa
L01	5	4	1	0	90	100	80	0.8**
L02	5	4	1	0	90	100	80	0.8**
L04	5	4	1	0	90	100	80	0.8**
L05	5	5	0	0	100	100	100	1***
L06	3	5	0	2	80	60	100	0.6*
L07	5	4	1	0	90	100	80	0.8**
L08	5	5	0	0	100	100	100	1***
L09	5	4	1	0	90	100	80	0.8**
L10	5	5	0	0	100	100	100	1***
L11	3	5	0	2	80	60	100	0.6*
L12	4	5	0	1	90	80	100	0.8**
L13	4	4	1	1	80	80	80	0.6*
L14	4	2	3	1	60	80	40	0.2 (ns)
**Overall**	**58**	**56**	**9**	**7**	**87.7**	**89.2**	**86.2**	0.59***

*P < 0.05

** *P* < 0.01

*** *P* < 0.001

ns = not significant.

**Table 3:** Conventional RT-PCR results by laboratory and statistical analyses of agreement. The concordance, specificity, sensitivity and K coefficient with p-value were calculated per each laboratory. Eleven laboratories (out of fourteen) submitted results for this part of the PT. Laboratories L03, L07 and L13 did not submit any results for the molecular test.

Laboratory	True positive	True negative	False positive	False negative	Concordance (%)	Sensitivity (%)	Specificity (%)	Kappa
L01	6	4	0	0	100	100	100	1***
L02	6	4	0	0	100	100	100	1***
L03	6	4	0	0	100	100	100	1***
L04	6	4	0	0	100	100	100	1***
L06	6	4	0	0	100	100	100	1***
L08	6	4	0	0	100	100	100	1***
L09	5	4	0	1	90	83.3	100	0.8**
L10	6	4	0	0	100	100	100	1***
L11	6	4	0	0	100	100	100	1***
L12	5	4	0	1	90	83.3	100	0.8**
L14	6	4	0	0	100	100	100	1***
**Overall**	**64**	**44**	**0**	**2**	**98.2**	**96.7**	**100**	0.93***

** *P* < 0.01

*** *P* < 0.001

### Direct fluorescent antibody test results

Overall, 92.9% (13/14) of laboratories submitted results by DFA test. Three laboratories (23.1%, 3/13) correctly identified all samples (K = 1 and 100% concordance). Seven laboratories (53.8%, 7/13) presented a decreased specificity due to the presence of FP, whilst five CVLs (38.5%, 5/13) failed to identify at least one rabies positive samples and presented a reduced sensitivity; two laboratories (15.4%, 2/13) submitted results presenting both FP and FN (Table 2, Fig 1). All but one p-value were below the 0.05 significance threshold level, meaning that the agreement between observed/expected results had not occurred by chance (Table 2). The average sensitivity was 89.2% with a range from 60% to 100%, whilst the average specificity was 86.2%, ranging from 40% to 100%. The mean concordance was 87.7% ranging from 60% to 100% (Table 2). Based on the acceptance criteria, results from eight laboratories out of thirteen (61.5%, 8/13) are considered acceptable.

The inter-laboratory agreement analysis showed that sample 10 (S10—Uninfected CNS lipofuscin synthetic RNA) presented the lowest level of inter-laboratory concordance (61.5%), while five laboratories (5/13) wrongly scored S10 as positive for rabies virus (Table 4). No significant association was found between the result obtained by RT-PCR and the misdiagnosis of S10 by DFA test. The inter-laboratory concordance for sample 9 (S9—Uninfected CNS lipofuscin) was 92.3%. Sample 1 (S1—Fixed RABV strain) and sample 2 (S2—RABV AFR2) presented an inter-laboratory concordance of 84.6% whilst sample 4 (S4—DUV) presented an inter-laboratory concordance of 76.9%. Amongst samples 6, 7 and 8 (uninfected CNS), the inter-laboratory agreement was 84.6%, 92.3% and 100%, respectively (Table 4).

**Table 4:** Overall performances. The table summarizes the composition of each sample, the expected results and the performances of participants. Expected results for S10 is negative by fluorescent antibody test but positive by molecular testing. Out of fourteen laboratories, thirteen (13/14) submitted results by the DFA test and eleven (11/14) submitted results by conventional RT-PCR.

		DFA				RT-PCR			
Sample	composition	Expected results	Positive	Negative	Inter-laboratory Concordance (%)	Expected results	Positive	Negative	Inter-laboratory Concordance (%)
S1	CVS-11	positive	11	2	84.6	positive	11	0	100
S2	RABV DOG AFR2	positive	11	2	84.6	positive	11	0	100
S3	RABV DOG EX-AFR1	positive	13	0	100	positive	11	0	100
S4	DUVV	positive	10	3	76.9	positive	11	0	100
S5	RABV HB AFR3	positive	13	0	100	positive	11	0	100
S6	Negative SNC	negative	2	11	84.6	negative	0	11	100
S7	Negative SNC	negative	1	12	92.3	negative	0	11	100
S8	Negative SNC	negative	0	13	100	negative	0	11	100
S9	Negative SNC with lipofuscin	negative	1	12	92.3	negative	0	11	100
S10	Negative SNC with lipofuscin and addition of synthetic RNA	negative	5	8	61.5	positive	9	2	81.8

### RT-PCR results

**Fig 2:**
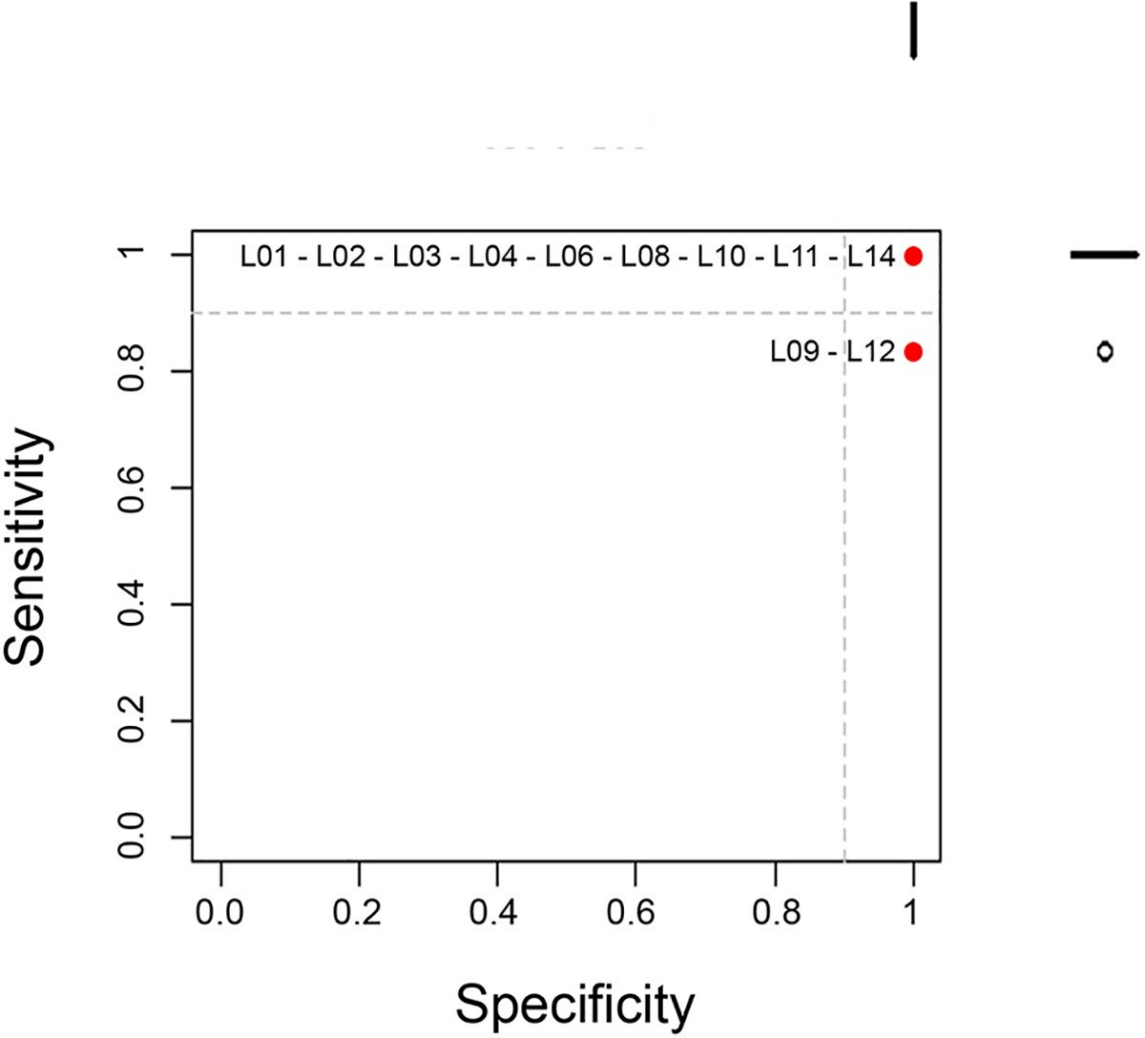
Scatter plot of sensitivity by specificity of RT-PCR submitted results. The sensitivity (y-axis) and the specificity (x-axis) are shown for each laboratory (red dots and laboratory ID). Nine laboratories out of eleven submitting results by RT-PCR correctly identified all samples. Those laboratories are found in the top right corner.

Overall, 78.6% (11/14) of laboratories submitted results using a conventional RT-PCR protocol recommended by the PT organizer [11]. Nine laboratories (81.8%, 9/11) correctly identified all samples (K = 1 and 100% concordance) while the other two wrongly identified one sample, thus presenting a substantial agreement (K = 0.8) (Table 3, Fig 2). Agreement occurring by chance was refuted since all K coefficient p-values were below the 0.05 significance threshold level. The average sensitivity of the test was 96.7%, ranging from 83.3 to 100%, while specificity was 100%, as no laboratories reported any FP using this test. The mean concordance was 98.2% ranging from 90% to 100% (Table 3). Based on the acceptance criteria, results from nine laboratories (9/11) are considered acceptable.

In regards to the inter-laboratory agreement, S10 (Uninfected CNS lipofuscin RNA) presented the lowest level of inter-laboratory concordance (81.8%), while the remaining samples presented an inter-laboratory concordance of 100% (Table 4). Critically, the stability control performed ruled out a possible degradation of the synthetic RNA, as the detection of the amplicon was unaffected either by storage at 37°C for two weeks or by long term storage for 6 and 12 months.

Overall, a higher concordance (98.2%) was observed by conventional RT-PCR rather than by the DFA test. Both the sensitivity (96.7% vs 89.2%) and specificity (100% vs 86.2%) were higher for RT-PCR than for DFA test (Tables 2 and 3).

### Technical questionnaire

Overall, 85.7% (12/14) of laboratories returned the technical questionnaire on GLPs and BB. However not all responding laboratories returned the questionnaire fully filled. Answers are presented in Table 5. The χ^2^ test was used to evaluate the significance of variables included in the questionnaire, which could potentially affect the results. Only the concordant/discordant results from laboratories responding to the questionnaire were included.

**Table 5:** Information on laboratory practices. Laboratories answered a questionnaire related to rabies facilities, virus identification by the DFA test or conventional RT-PCR and biosafety measures. Twelve out of fourteen (12/14) laboratories returned the questionnaire, although not all sections were filled in some of them. For relevant factors, association test was applied to statistically evaluate any association with results agreements. None of the evaluated factors were significant (pχ^2^>0.05).

Rabies facilities						
12 laboratories provided answers						
Facilities equipped for (more than one answer possible)	DFA test	RT-PCR	Viral isolation	others		
Number of laboratories (%)	11 (92%)	10 (83%)	0	0		
Identification of rabies virus by DFA test						
11 laboratories provided answers						
Slide preparation	smear	print	both	pχ^2^ = 0.74		
Number of laboratories (%)	6 (55%)	2 (18%)	3 (27%)			
Results in disagreement	2 FP—5 FN	1 FP	2 FP			
Number of slides prepared / sample	1	2	>2	pχ^2^ = 0.37		
Number of laboratories (%)	1 (9%)	7 (64%)	3 (27%)			
Results in disagreement	2 FN	4 FP—2 FN	1 FP - 1FN			
Fixation method	Acetone	Heat	both			
Number of laboratories (%)	11 (100%)					
Results in disagreement	5 FP—5 FN					
Fixation time	30 mins	60 mins	4 hours	Overnight	pχ^2^ = 0.91	
Number of laboratories (%)	4 (36%)	5 (46%)	1 (9%)	1 (9%)		
Results in disagreement	2 FP—2 FN	2 FP—3 FN	1 FP			
Fixation temperature	RT	-20	pχ^2^ = 0.63			
Number of laboratories (%)	1 (9%)	10 (91%)				
Results in disagreement	1 FP	4 FP—5 FN				
Use of commercial conjugate	BIORAD	FUJIREBIO	Commercial (no information)		Non commercial	pχ^2^ = 0.18
Number of laboratories (%)	7 (64%)	1 (9%)	2 (18%)		1 (9%)	
Results in disagreement	3 FP—1 FN	1 FP	4 FN		1 FP	
Rinsing solution	PBS	Water	Both			
Number of laboratories (%)	11 (100%)	0	0			
Results in disagreement	5 FP - 5FN					
Number of washes (of 5 mins)	1	2	3	pχ^2^ = 0.53		
Number of laboratories (%)	0	7 (64%)	4 (36%)			
Results in disagreement		3 FP - 3FN	2 FP - 2FN			
Mounting medium	Commercial	Non commercial	pχ^2^ = 0.58			
Number of laboratories (%)	9 (82%)	2 (18%)				
Results in disagreement	3 FP + 5 FN	2 FP				
Mounting medium pH	8	8.5	9	9.5	NP	pχ^2^ = 0.59
Number of laboratories (%)	1 (9%)	2 (18%)	1 (9%)	2 (18%)	5 (46%)	
Results in disagreement		1 FP - 2FN		1 FP	3 FP - 3FN	
Number of readers	1	2	>2	pχ^2^ = 0.47		
Number of laboratories (%)	0	4 (36%)	7 (64%)			
Results in disagreement		1 FP—2 FN	4 FP—3 FN			
Identification of rabies virus by conventional RT-PCR						
10 laboratories provided answers						
technique routinely used	Yes	No	pχ^2^ = 0.16			
Number of laboratories (%)	4 (40%)	6 (60%)				
Results in disagreement	2 FN					
8 laboratories provided answers						
RNA extraction						
Time extraction	during slides preparation		after slides preparation		pχ^2^ = 0.24	
Number of laboratories (%)	7 (87.5%)		1 (12.5%)			
Results in disagreement	1 FN		1 FN			
Extraction methods	Kit QIAGEN	Kit Macherey-Nagel		pχ^2^ = 0.75		
Number of laboratories (%)	7 (85.7%)	1 (12.5%)				
Results in disagreement	2 FN					
Elution volume	30μl	50μl	60μl	pχ^2^ = 1		
Number of laboratories (%)	2 (25%)	4 (50%)	2 (25%)			
Results in disagreement		1 FN	1 FN			
Biosafety & Sampling						
11 laboratories provided answers						
Sample collection (more than one answer possible)	Scull	Occipital	Both			
Number of laboratories (%)	6 (55%)	2 (18%)	3 (27%)			
Area of CNS used (more than one answer possible)	Hippocampus	Medulla	Cerebellum	Cortex	Spinal cord	not specified
Number of laboratories (%)	9 (100%)	5 (55.6%)	7 (77.8%)	5 (55.6%)	2 (22.2%)	1 (11.1%)

Sample preparation						
Biosafety cabinet type II	Yes	No				
Number of laboratories (%)	9 (82%)	2 (18%)				
Disinfectant (more than one answer possible)	Sodium hypochlorite				Virkon	Ethanol
concentration	0.5%	1%	5%	10%	1%	70%
Number of laboratories (%)	2 (18%)	3 (27%)	1 (9%)	1 (9%)	7 (64%)	4 (36%)

FP: false positive, FN: false negative.

#### Rabies facilities

Twelve laboratories provided answers relating to the equipment available for rabies diagnosis. Eleven laboratories (91.7%, 11/12) are equipped to perform the DFA test, while ten (83.3%, 10/12) have the equipment for conventional RT-PCR testing (Table 5).

#### Identification of rabies virus by DFA test

Eleven laboratories included information about the methodologies applied for the routine diagnosis of rabies outside of the PT. Regarding the DFA test, 54.5% of the laboratories (6/11) use the smear method to prepare slides, 18.2% (2/11) use the print method and 27.3% (3/11) use both techniques. When rabies cases are suspected or animal bites are involved, three of the laboratories (27.3%, 3/11) prepare at least three slides per sample, seven (63.6%, 7/11) prepare two slides per sample and one laboratory (9.1%, 1/11) only prepares one slide. Although all laboratories (100%, 11/11) use acetone as a fixative, the fixation time varies, ranging from 30 minutes (36.4%, 4/11), to one hour (45.5%, 5/11), to four hours (9%, 1/11) to overnight (9%, 1/11). All laboratories but one (9%, 1/11) proceed with fixation at -20°C. Information collected on fluorescent conjugate showed that 90.9% (10/11) of laboratories stain the samples by using commercially produced conjugates (63.6% anti-rabies nucleocapsid conjugate from Bio-Rad (Cat No. 3572112), 9% FITC Anti-Rabies Monoclonal Globulin from Fujirebio (Cat No. 800–092), 18.2% unknown). Five laboratories provided information on conjugate reconstitution and working dilution, they all followed the manufacturer’s instruction. The laboratory which used a non-commercial conjugate did not provide any information on either the source or the method of production of the reagent. PBS (pH not specified) is used by all laboratories as rinsing solution for either two (63.6%, 7/11) or three (36.4%, 4/11) washes of 5 minutes. Information collected on the type of mounting medium showed that 81.8% (9/11) of laboratories used a commercially sourced cover-glass mounting medium. Great variations were observed regarding the pH of mounting medium; 9% (1/11) declared a pH of 8, 18.2% (2/11) declared a pH of 8.5, 9% (1/11) declared a pH of 9 and 18.2% (2/11) declared a pH of 9.5. Almost half of the laboratories (45.4%, 5/11) were not able to specify the pH of the mounting medium solution used. The number of readers was at least two for all laboratories (Table 5). No significant association was observed between laboratory performance and any of the reported variations in the technique (all p-values > 0.05, Table 5).

### Identification of rabies virus by conventional RT-PCR

Four laboratories declared to have routinely applied RT-PCR testing even before this PT was organised. Of the 8 laboratories that filled the questionnaire in relation to the technicality of the molecular testing protocol, seven (87.5%, 7/8) proceeded with nucleic acid extraction in parallel with DFA test slide preparation. All laboratories extracted RNA using commercial kits according to the manufacturer’s instructions; among these, seven laboratories (87.5%, 7/8) used an extraction kit from QIAGEN (Cat No. 74106) and one laboratory performed RNA extraction using a kit from Macherey-Nagel (Cat No. 740955). The elution volume varied greatly among laboratories, ranging from 30μl (25%, 2/8), to 50μl (50%, 4/8), to 60μl (25%, 2/8) (Table 6), likely due to variations in the kit instructions–i.e. 30 to 50 μl in the case of QIAGEN and 60 μl for Macherey-Nagel. Similar to the DFA test methodologies, no change in any of these steps had a significant influence on laboratory performance (all p-values > 0.05, Table 5).

**Table 6:** Information on routine diagnostic activities. The technical questionnaire reported information on the diagnostic activities carried out between January 2016 and September 2017. The number of samples from both domestic and wildlife animals and the confirmed rabies positive cases were communicated. Based on the quantity of samples analysed monthly, a weighted K was calculated.

Laboratory	Number of domestic animal samples analysed	Number of positive domestic samples	Number of wildlife animal samples	Number of positive wildlife samples	Number of samples analysed /month	Samples positive for rabies (%)	weighed Kappa
**L01**	351	314	4	2	17.8	89	0.869**
**L02**	30	27	285	10	15.8	12	0.869**
**L04**	25	21	1	0	1.3	81	0.905***
**L05**	574	363	8	6	29.1	63	1***
**L06**	105	87	1	0	5.3	82	0.636**
**L07**	176	131	5	2	9.1	73	0.887***
**L08**	41	39	3	1	2.2	91	1***
**L09**	13	10	0	0	0.7	77	0.905***
**L10**	26	19	0	0	1.3	73	1***
**L11**	34	19	9	8	2.2	63	0.636**
**L12**	0	0	0	0	0	0	0.810**

** *P* < 0.01

*** *P* < 0.001.

#### Biosafety & Sampling

Eleven of the questionnaire filled in by the laboratories provided details on biosafety issues. The information collected showed that sample collection was more commonly achieved by opening the skull (54.6%, 6/11) than via the occipital route (18.2%, 2/11) with the help of a pipette or straw. Three laboratories (27.3%, 3/11) alternate the techniques and apply both methods of collection. More specifically, the nine laboratories applying the opening of the skull method preferably target the hippocampus (100%, 9/9) and the cerebellum (77.8%, 7/9), whilst the cortex (55.6%, 5/9), medulla (55.6%, 5/9) and spinal cord (22.2%, 2/9) are less frequently selected. Nine laboratories (81.8%, 9/11) process rabies suspected samples under a type-2 biosafety cabinet. Finally, the questionnaire demonstrated great variations among laboratories in the choice of disinfecting solutions used during necropsy and in the laboratory. In particular, one disinfectant is used for both facilities and equipment in 36.4% (4/11) of laboratories, while 63.6% (7/11) of laboratories use a combination of either two or three solutions. Sodium hypochlorite at various concentrations and Virkon [1%] are the most widely used disinfectants in 7/11 laboratories (63.6%), whilst 36.4% declared to use ethanol (70%) (Table 5).

#### Diagnostic sample analysis

Finally, eleven laboratories provided information on their diagnostic workload between January 2016 and September 2017, with three laboratories analysing more than fifteen samples per month, two laboratories analysing between 5 to 15 samples and six laboratories analysing less than 5 samples per month (Table 6). This information was used to define the weighted K as presented in Table 6. Critically, the number of samples analysed per month showed no influence on laboratory performance (pχ^2^ = 0.57). The questionnaire gave some insights into the burden of rabies in each country. In particular, most laboratories reported 63% to 91% of analysed field samples as being positive for lyssaviruses; on the other hand, only 12% of samples were positive in the L02 laboratory (Table 6). However, results from L02 laboratory showed great variation between samples collected from wildlife and from domestic animals. In particular, a large number (285) of samples were collected from wildlife, of which only 3.5% tested positive for rabies virus, while fewer samples (30) were collected from domestic animals, out of which 90% tested positive for rabies (Table 6).

## Discussion

We herewith describe the results of an inter-laboratory trial for rabies diagnosis in animals, which included thirteen Veterinary Laboratories and one Public Health Institute from West, Central and Eastern Africa. The general aim of this study was the implementation and evaluation of the rabies diagnostic capabilities of the selected laboratories, which were more specifically the assessment of DFA test performances and the implementation of a molecular test.

In agreement with previously published data [13], the overall statistical analysis demonstrated that the highest proportion of concordant results was obtained by using conventional RT-PCR (98.2%) and that sensitivity (96.7%) and specificity (100%) were both higher than those obtained by DFA test (89.2% and 86.2%, respectively). However, with respect to a previous inter-laboratory study [13], the highest reproducibility of results of the RT-PCR test might be partly due to the fact that all laboratories applied the recommended protocol [11] for molecular testing, although with some variations (Table 5), compared to the DFA protocol. According to the acceptance criteria, the overall performance of seventy nine percent (79%, 11/14) of participating laboratories was considered as favorable.

Regarding the performance of the DFA test, which at the time of the PT exercise was the gold standard technique indicated by OIE, our trial showed eighty-eight percent (87.7%) concordance in the African setting. Compared to results from other PT trials [13] [14], the performances of African laboratories in the diagnosis of rabies cases in animals through the DFA test are to be considered as highly acceptable. However, differences in the strains used and in panel preparation prevent a direct comparison of trials. The panel composition of our PT was designed to focus on relevant rabies strains circulating in the African dog population rather than on rarely reported lyssaviruses associated to bats or other wildlife. Conversely to what had been organized for the PT test exercise involving Latin American countries and the Caribbean [14], the PT prepared by the FAO RC for rabies included the assessment of the entire diagnostic procedure, including slide preparation and fixation method.

The PT panel was composed of 4 RABV, 1 African bat lyssavirus and 5 negative samples including a sample spiked with a synthetic RNA. The material was freeze dried to ensure a higher stability, particularly during the shipment. As a matter of fact, the lyophilisation of fresh brain specimen is standardly applied by reference laboratories as the most appropriate compromise between clinical samples and laboratory produced material [13][28][29]. Furthermore, similarly to other PT exercises, laboratories received a unique set of coded vials to which both tests were to be applied to match the reality of routine diagnosis [13]. Upon reception of suspected samples, laboratories usually apply the different methods available at their facilities, and results between tests can differ based on technical sensitivity but also on the detection target with respect to each diagnostic testing procedure.

The performance of molecular testing by means of conventional RT-PCR showed that the recommended protocol [11] was well applied. Overall, two false negative results were identified. In addition, the use of RT-PCR as a confirmatory test allowed four out of five laboratories (4/5) to detect false negative results obtained through the DFA test. The same applied to the nine false positive samples identified by DFA test, which were not confirmed through PCR. These observations further validate the importance for laboratories to have access to more than one recommended technique, in particular for targeting both viral antigens and the genome. Importantly, the molecular protocol was successfully introduced to six laboratories whose questionnaires confirmed they had never used this approach for rabies diagnosis before the PT, despite possessing the equipment and competencies. This demonstrates that molecular approaches can be easily implemented across Africa through training efforts, such as the ones already implemented for Avian Influenza and other priority diseases [8][30]. Of note, although at the time this proficiency test was organized molecular testing could only be used as a confirmatory test, in May 2018 the OIE released an update of the Manual of Diagnostic Tests and Vaccines for Terrestrial Animals where dRIT as well as conventional and real-time RT-PCR are recognized as gold standard techniques for the diagnosis of rabies (5). This is particularly relevant for African laboratories, for which the regular maintenance of equipment can easily become a huge obstacle for routine diagnostic procedures. Consequently, the availability of more than one gold standard techniques is predicted to reduce the risk of diagnostic activity suspension. Importantly, this study does not claim the superiority of a molecular-based over an antigen-based approach to detect rabies virus, but emphasizes the need to access several methodologies complementary to each other.

Other than proving the efficacy of molecular tests in determining positive rabies cases, the PT allowed to evaluate the pros and cons of this approach compared to antigen-based methods. Results confirmed a higher risk of misinterpretation by DFA test, with the presence of nine false positive that were all scored negative by RT-PCR. Interestingly, six out of nine (6/9) FP involved negative samples containing lipofuscin, a golden pigment which can be naturally found in the brain of aging animals [18] and impair the accuracy of inexperienced slide readers. These results confirm that a correct interpretation is a main challenge when performing the DFA test, particularly in the case of weak or non-specific florescence compared to the band visualization of RT-PCR results, which minimises the requirement for personal observation.

Another advantage of the molecular approach is a better sensitivity in case of putrefied samples, which are often unfit for the detection of viral antigens [31]. This is an important factor in the African setting, where the arrival of field samples to diagnostic facilities can be delayed due to transportation issues, samples can be incorrectly stored [10] or suspected animals can be exhumed several weeks after their burial following suspicion of human rabies cases [32]. Although real-time RT-PCR protocols that detect smaller fragments is a better option for detection of the viral genomes in case of advanced decomposition [32], a recent study from India [31] showed that out of thirty six [36] field putrefied samples unfit for detection of viral antigens by either DFA test or dRIT, thirty five [35] of them still tested positive by mean of a well-known hemi-nested RT-PCR also targeting rabies virus N gene [33]. In any case, although opting for molecular testing increases the possibility to identify the virus in putrefied material, it is not exempt from misdiagnosis. Therefore, negative results of compromised samples should always be carefully interpreted.

Nevertheless, the main drawback of PCR approaches is the elevated risk of cross-contamination, in particular during the necropsy of carcasses, although contamination between samples remains high during the whole procedure. Thus, laboratory technicians need to be properly trained and also adequately informed not only on the process of virus inactivation but also on nucleic acid degradation. A study from *Aiello et al* assessing the efficacy of various disinfection protocols showed that none of the tested disinfectants proved to be effective when following label instructions and, in particular, when molecular methods were applied [34]. This study clearly indicates that, although virus inactivation is rapidly obtained, the complete elimination of any nucleic acid residues requires the application of disinfectant for a longer period of time, e.g. Virkon powder at 1% for 60 minutes or sodium hypochlorite at 3% for 30 minutes [34]. Thus, any facilities applying molecular testing should carefully validate a disinfection protocol before introducing molecular techniques and implement a procedure to go back to uncontaminated original material in the case of discordant results from molecular versus antigen-based methods, especially for fresh diagnostic samples, for which similar results are expected.

A novelty of this PT was that a negative sample spiked with a synthetic RNA was included in the panel. Whilst this sample could be interpreted to mimic degraded samples unfit for DFA test, it could also be considered as a contaminated sample. This sample was also prepared with brain tissues containing lipofuscin and although another negative sample also presented non-specific florescence ascribed to the presence of lipofuscin, the inter-laboratory concordance between the two showed variations by DFA test. Indeed, the inter-laboratory concordance by DFA test for the synthetic RNA spiked sample and the synthetic RNA free, aspecific sample was 61.5% and 92.3%, respectively. Although, at first, one could suggest that the results obtained by molecular testing may have misguided a handful of readers, no significant correlation could be established between the results of each tests. Furthermore, out of 16 false results (7 FN and 9FP) reported using DFA, 13 were also tested using RT-PCR and correctly identified. Importantly, laboratories did not correct the results of the DFA test, clearly showing that the results by means of molecular testing did not influence the results obtained by immunofluorescence. Importantly, FP results were given by samples both containing and non-containing lipofuscin, 6 and 3, respectively. These results can be due to various issues such as manipulation of the vials, preparation of the slides and/or reagents, result interpretation and/or reporting. The difficulties encountered by participants with the sample spiked with RNA were expected by the provider. Nevertheless, although more difficult to handle, this sample mirrors the reality of field situation. In any case, results analysis of the DFA test excluding this sample did not significantly affect the overall results (S1 Table).

Among the disadvantages of molecular tests, several authors have suggested that there is a variable efficacy of protocols according to the strain being diagnosed and a lack of detection of divergent LYSVs [35]. Results from this PT confirmed that the protocol suggested for this trial was able to detect rabies strains and at least one LYSV species found in Africa. In addition, the RT-PCR adopted by participating laboratories targets a conserved region of the LYSV nucleoprotein gene and has been shown to cover the whole genus *Lyssavirus* in silico and *in vitro* [11]. In addition, the protocol allows for the amplification of 600 base pairs, which have been shown to be significant for phylogenetic classification of circulating strains through sequencing [11]. Unfortunately, we could not test laboratory performances in detecting most divergent LYSVs mainly due to safety reasons. Indeed, the vaccine protection for LYSVs belonging to phylogroups 2 and 3 is still under debate [36]. In this context, molecular protocols also have the advantage of early inactivation of diagnostic samples following immersion in lysis buffer or using tools like LFDs [37][38] or FTA cards [39] which both allow the preservation of viral nucleic acid and inactivate the virus [38][40]. In addition, sample typing will eventually provide important information on the source of the rabies case to local stakeholders. Ultimately, the transport of RT-PCR products to external sequencing services or Reference Laboratories does not qualify as a dangerous goods shipment, reducing the cost, the logistics and the risk associated with shipment of hazardous material. Although this PT did not include the further molecular characterisation of the viruses, the outsourcing of sequencing facilities will be included in future PT exercises to rule out possible contamination and to characterise the viral strains.

PT organisation activities included the collection of vaccination coverage datasheets from all participants. Amongst fourteen [14] laboratories, five (35.7%, 5/14) did not present satisfactory vaccine coverage. In a context of unstable political and economic climates, African laboratories are subject to frequent turnover of laboratory staff. Although, WHO recommends the pre-exposure vaccination to people at high risk such as laboratory staff working with rabies and rabies suspected samples, veterinarians, animal handlers and wildlife officers, newly recruited laboratory employees in Sub-Saharan Africa often have to financially cover for their own vaccination due to the lack of public funding [41][42]. The absence of control and obligation to vaccinate staff prior to accessing potentially rabies-exposed facilities, together with financial limitations, lead to the breach of this essential biosafety requirement [43]. Thus, this proficiency test also constituted a chance to update the vaccination status of all laboratory staff involved in rabies diagnostics and provided assistance in accessing the appropriate pre-exposure to non-protected laboratory staff. Vaccination procedure was facilitated by FAO and proof of completion of the whole vaccination protocol was requested before the shipment of panels. In addition, we suggest that the organization of regular PT exercises might be critical to the update of the vaccination status of lab staff across African laboratories, limiting a potential exposure to the deadly virus especially for newly recruited staff.

Another goal reached by the PT, as described, was to provide rabies control samples to African laboratories to be used as positive or negative controls for routine diagnostic activities or, more importantly, for in house validation procedures. This included both internal PT controls and blinded samples included in the panels, for which full information on the composition was provided by organizer after submission of the results. Among these, the uninfected tissue spiked with synthetic RNA is described for the first time in this study and shows promise in enhancing rabies diagnostics in African settings, by broadening chances for testing to facilities with limited resources, low biosafety measures and/or low vaccine coverage of staff. Upon submission of samples already inactivated (lysis buffer, LFDs, FTA cards), one could foresee, rabies diagnostic facilities free of infectious material in a laboratory solely equipped to perform molecular diagnostic.

The questionnaire revealed several technical variations particularly in the application of the DFA test protocol. Although studies reporting that outcomes of the DFA test can be significantly affected by various factors such as the fluorescent conjugate [28], the cover glass mounting medium and its associated pH [44], and also, the microscope and type of filters in used [45], we did not observe any influence of technical steps for both DFA test and RT-PCR, very likely due the low number of discordant results. Information regarding the maintenance of equipment, the type of fluorescent microscope and filters in use were not collected during this PT. Further improvements of the questionnaire for the future PT exercises will allow a more precise assessment of the effectiveness of the equipment. Interestingly, the only factor significantly affecting performances of the PT was the timing for the submission of results. Indeed, whilst eleven laboratories (11/14, 78.6%) respected the 28 days deadline posed by the organizer, three laboratories (3/14, 21.4%) submitted PT results after the allocated time period. Although stability control performed after 14 days at 37°C and 12 months at -20°C showed that all samples were still fit for both DFA test and RT-PCR, we cannot rule out improper storage in participating laboratories due to frequent power cuts. In any case, degradation of the samples could only explain FN results, while other causes lead to FP, such as bacterial growth which is excluded due to sample lyophilisation. Nevertheless, the occurrence of bacterial contamination is a common phenomenon which results in a characteristic nonspecific fluorescence and should easily be discriminated from a true fluorescence. Despite the fact that two out of these three laboratories did not respond to the questionnaire, information collected via email exchange suggested that regular interruptions in the diagnostic activity, a recent restructuring of laboratories and the recruitment of new staff members are most likely responsible for low DFA test performances in the PT. Thus, a delay in responding to this PT is mainly a reflection of the constrained situation faced by the concerned laboratories.

In any case, technical support from reference laboratories is key to ensure access to reference material, and to train laboratories for an appropriate application of recommended procedures. This can be achieved by participation in training or workshops, a solid communication among laboratories in regards the implementation of newly validated methods and regular participation in proficiency testing exercises. Since the completion of this proficiency test, a practical training for three laboratory technicians from one participating laboratory showing low performances during the PT was organized. This workshop investigated reasons behind the frequency of FN in this lab, enabled the identification of gaps in the protocol applied and pinpointed issues with the conjugate used for DFA test, i.e. the use of a non-validated home-made conjugate. The participation in another proficiency test would be critical to confirm that the protocol is now appropriately applied.

Finally, our PT was also used to collect information regarding routine diagnostic activities performed by the participant laboratories. Frighteningly, African laboratories reported a high percentage of positive cases ranging from 63% to 91%, which is particularly worrying also considering that 35.7% of laboratories did not present satisfactory vaccination cover prior to the exercise. Although rabies is endemic and has been listed as a priority disease in all participating countries (2), we found variable effort in rabies surveillance, with the number of samples analyzed per month ranging from one to more than fifteen. Interestingly, we found no association between routine diagnostic activities and PT performances for DFA test, suggesting that there is also a satisfactory maintenance of technical skills in laboratories with lower diagnostic activity.

In conclusion, the present study showed that veterinary African laboratories have the potential to provide reliable diagnostic for rabies, supporting the implementation of surveillance and control plans expected over the coming years, as countries work towards the elimination of rabies by 2030. However, the low levels of biosafety and vaccination coverage represent a serious concern, posing a risk to laboratory staff. In this context, we suggest that molecular techniques offer an appealing approach by showing higher sensitivity and specificity and a lower manipulation of hazardous material compared to antigen-based methods. In addition, the involvement of laboratories across Africa that previously received training on molecular techniques for other animal diseases would allow for rapid enhancement of rabies surveillance, also reducing the time and costs related to the transport of samples to facilities equipped for DFA test. In this context, it is critical that the economic support provided by international organizations is associated with technical support, continuous training and periodic assessment of performance, in addition to the promotion of biosafety measures, including the vaccination of new staff. In any case, the organization of future PT programs is also necessary to substantiate the conclusions of this study, due to the small, albeit acceptable number of participating laboratories.

## Supporting information

S1 Appendix General instructions to perform the PT. (PDF)

S2 Appendix Protocols of the DFA test and conventional RT-PCR. (PDF)

S3 Appendix Questionnaire. (PDF)

S1 Table Analysis of the Fluorescent antibody test results by laboratory excluding sample S10. The concordance, specificity, sensitivity and K coefficient with p-value were calculated omitting sample S10. Compared to the results analysis including all ten PT samples, the concordance, the specificity and the kappa, respectively increased from 87.7% to 90.6%, 86.2% to 92.3% and 0.59 to 0.66. No statistically significant differences between the concordance, the specificity and the kappa including or excluding sample S10 were established. *P < 0.05; ** *P* < 0.01; *** *P* < 0.001; ns = not significant.
(PDF)
