# Supplementary material for: An inter-laboratory trial as a tool to increase rabies diagnostic capabilities of Sub-Saharan African Veterinary laboratories
Source: PLoS Negl Trop Dis. 2020 Feb 10;14(2):e0008010. doi: 10.1371/journal.pntd.0008010 (PMC7010240; doi:10.1371/journal.pntd.0008010)
Supplement: S1 Appendix — (PDF) [file pntd.0008010.s001.pdf]

GENERAL INSTRUCTIONS

The panel includes:
- 1 negative control;
- 1 positive control;
- 10 unknown samples (lab code – VIAL 1/10).

All samples are freeze-dried, although **they may contain infectious material.**

Mandatory requirement: ALL OPERATORS MANIPULATING SAMPLES FROM THE RABIES PT PANEL MUST BE FULLY VACCINATED (3 SHOTS) AGAINST RABIES VIRUS.

Here below you may find some useful guidelines for the correct handling of the samples:

- Upon arrival, open the second container of the parcel under a biosafety cabinet;

- Verify that the samples have reached your laboratory intact; the FAO Reference Centre for rabies (RC) must be immediately informed in case any damage has occurred;

- If testing is not conducted immediately, store the panel at ≤ -18°C;

- re-suspend the content of each vial with 1.0 ml of deionised sterile water, by using a syringe and without opening the cap;

- The procedure should be carried out by starting with the negative control, followed by the unknown samples (from 1 to 10) and finally the positive control. The syringe has to be changed at each sample;

- Leave the samples under the biosafety cabinet at room temperature (average +24°C) for 30 minutes prior to testing to allow the samples to rehydrate, and mix each vial content by gentle rotation every 15 minutes;

- Analyse the PT samples using the direct fluorescent antibody testing protocol routinely applied and using the conventional RT-PCR recommended by the IZSVe. Primers have been supplied and if they have not been received, please contact Morgane Gourlaouen. For your information or in case of any doubts regarding the procedures, protocols are also provided.

- Pay particular attention to avoid cross-contamination among samples. Change the second (outer) pair of gloves before starting to process the next vial (once cross-contamination of samples occurs, PT results will be inevitably jeopardized);

- It is recommended to prepare the samples for the confirmation test in parallel, when preparing the slides for the test by immunofluorescence. If it is impossible to extract RNA directly, samples must be kept in the freezer (≤ -70 °C) until the time of extraction.

- In case more than one person carries out the analyses, please make sure that at least one operator completes the procedure for all the samples, controls included;

- Interpretation of the slides should be preferentially conducted by two operators;

- Once re-suspended, store the remaining material at ≤ -70°C for further testing;

- Once you receive the final feedback from the RC, you may use the PT samples as reference material for further direct fluorescent antibody testing.
