# Supplementary material for: An inter-laboratory trial as a tool to increase rabies diagnostic capabilities of Sub-Saharan African Veterinary laboratories
Source: PLoS Negl Trop Dis. 2020 Feb 10;14(2):e0008010. doi: 10.1371/journal.pntd.0008010 (PMC7010240; doi:10.1371/journal.pntd.0008010)
Supplement: S2 Appendix — (PDF) [file pntd.0008010.s002.pdf]

1- Fluorescent Antibody Test (FAT)

1.1 Reconstitution of the vials

Reconstitute each vial by adding 1 ml of sterile deionised water (follow general instruction form). It is important to change the outer gloves and syringes between samples and to start with the reconstitution of the negative control, followed by the coded samples and to finish with the positive control.
Allow the vials to sit for 30 mins at room temperature for rehydration of the lyophilised brain sample.

1.2 Preparation of test slides:

Using pipette tips, mix and collect some sample. Prepare 2 slides for each coded sample.
Each step of the manipulation must be carried out under **Biosafety cabinet class II**.

1.3 Fixation

- Once dried, both test slides and positive/negative control slides are immerse in slide racks (glass container) filled with cold pure acetone and fixed for at least 30 minutes.

Hellendhal staining jar

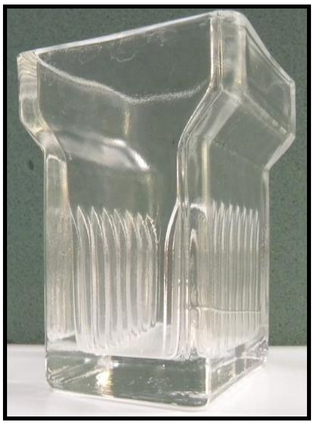

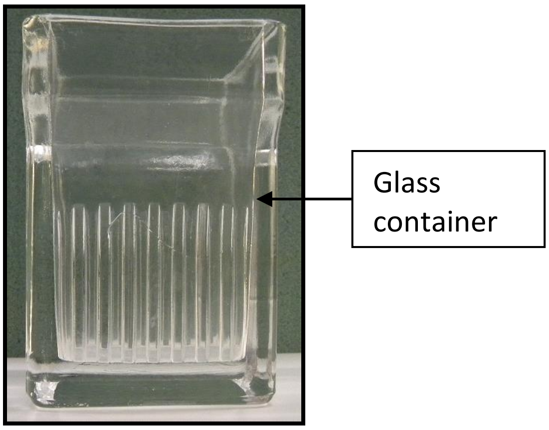

N.B: The positive control should be fixed in separate containers to avoid transfer of tissues from sample slides to the positive control during fixation.

- Remove the slides from acetone and air-dry the slides at room temperature for 15/20 minutes (under chemical cabinet).

1.4 Immunofluorescent staining

- Calculate the total volume of the antibody you need multiplying the number of slides that will be analysed by the amount that will be distributed on each slide (we recommend to use between 20 µl

to 50 µl). (Number of slides x volume (µl) of antibody per slide= total volume (µl) of antibody needs for the work session).

To this volume, add the required amount of Evan's blue in order to obtain 1:20 dilution.

(For example, for 100 µl total volume of antibody, 5 µl of Evan's blue at 1% will be added).

- Add 20 µl to 50 µl of conjugate and Evan's blue on each slide. Incubate the slides for 30 minutes at 37°C in a humidified chamber to avoid drying of the conjugate.

1.5 Washing

- After incubation, slides are washed twice with PBS (5 min for each wash) and once with distilled water for a few seconds. Drain the slides on filter paper.
- When dry, one drop of mounting medium is added on each slide. Mounting medium is a solution of 20% glycerol in 50 mM Tris buffer (supplemented with NaCl 150 mM) pH 9.6.
- Carefully place the coverslip avoiding the formation of bubbles.

1.6 Microscope reading

The procedure consists in allowing a labelled antibody (fluorescein isothiocyanante) to react with a specific antigen (if present) and in observing the reaction under a fluorescence microscope.

2- Confirmatory test – conventional RT-PCR
Rabies virus Gene N

It is recommended to prepare the samples for the confirmatory test by mean of conventional RT-PCR in parallel with the slides for immunofluorescence and to extract the RNA as soon as it is possible.
In case the RNA extraction does not occur directly after sample preparation, tubes must be kept at -80°C.

2.1 Sample preparation

Following the general instruction guide, suspend all lyophilized samples with 1 ml of sterile distilled water.
Samples for molecular confirmatory diagnostic test are 1:10 SNC material diluted in Phosphate Buffer Saline (PBS). We recommend to collect 100 μl of sample and to add 900 μl of sterile PBS. Mix thoroughly the SNC homogenates by vortexing or using disposable stirrer like a 1 ml pipette tip. Use a sterile clean tip for each sample, open only one tube at the time and change gloves between each samples, the risk of cross-contamination is high at this step.
Samples are then centrifuged for 15 minutes at 4°C at 3,000 g. At the bottom of the tube, a pellet of SNC material should be visible. The supernatant is the starting material for the confirmatory test. According to your RNA extraction protocol, collect the necessary volume of sample and proceed with extraction. Do not forget to include a negative control for the extraction step (Bext).
Once RNA is extracted, reaction mix for conventional RT-PCR can be prepared.

2.2 Conventional RT-PCR

The primers used for the detection of rabies by conventional RT-PCR are: primer RabForPyro (forward primer), RabRevPyro 1 (reverse primer 1), RabRevPyro 2 (reverse primer 2), RabRevPyro 3 (reverse primer 3). Resuspend the primers with adequate volume of TE buffer or RNase free water to obtain a concentration of 100 μM (follow manufacturer's instruction).

Dilute the primers 1:10 in TE buffer or RNase free water to a final working concentration of 10 μM as explained below:

- Prepare the forward primer mix: pipette 10 μl of the 100 μM dilution RabForPyro and add 90 μl of TE buffer or RNase free water.

- Prepare the reverse primers mix: pipette 10 μl of the 100 μM dilution RabRevPyro 1, 10 μl of the 100 μM dilution RabRevPyro 2, and 10 μl of the 100 μM dilution RabRevPyro 3 and add 70 μl of TE buffer or RNase free water.

Prepare the RT-PCR mix according to the protocol recommended by the IZSVE (see below), do not forget to include a negative control for the RT-PCR mix (Bmix).

Conventional RT-PCR products can be analysed using agarose gel.

One Step RT-PCR Rabies gene N

Mix Preparation

Defreeze the reagents. Prepare the following mix (table below) in a sterile Eppendorf-type tube. Carefully mix the reagents then vortex and spin for few seconds. It is important to keep the enzymes refrigerated during the whole process. Do not forget to include a negative control for PCR mix (Bmix) and a positive control.

REAGENTS NAME	COMPANY/CODE	STORAGE CONDITION
**Primer RabPyro Forward**<br><br>5' – AACACYYCTACAATGGA – 3'	Eurofins	-20° C (+2-10)° C
**Primers RabPyro Reverse (3 primers)**		
5' - TCCAATTNGCACACATTTTGTG – 3'	Eurofins	-20° C (+2-10)° C
5' – TCCARTTAGCGCACATYTTATG – 3'	Eurofins	-20° C (+2-10)° C
5' – TCCAGTTGGCRCACATCTTRTG – 3'	Eurofins	-20° C (+2-10)° C
**OneStep RT-PCR Kit** (PCR buffer 5X, dNTPs mix)	Qiagen<br>cod. 210212	-20° C (+2-10)° C
**RNase Inhibitor** 40 U/µl	Promega<br>cod. N2611	-20° C (+2-10)° C

REAGENTS/STOCK CONCENTRATION	FINAL CONCENTRATION	µL X 1 REACTION	µL TOTAL
RNase- free water	/	13.75	
Primer RabPyro Forward mix 10µM	400 nM	1	
Primers RabPyro Reverse mix 10µM	400 nM	1	
PCR Buffer 5X	1X	5	
dNTPs mix 10mM	0.4 mM	1	
RNase Inhibitor 40 U/µl	20 U	0.25	
One Step RT-PCR Enzyme Mix	/	0.5	
REAGENT VOLUME		22.5µl	
RNA		2.5µl	
FINAL REACTION VOLUME		25µl	

Distribute the mix (22.5 µl) in sterile PCR tubes or in 96 well plates and add RNA to appropriate labeled tubes or well. Place lids on tubes or adhesive cover on 96 well plates. Read using the appropriate thermal profile.

Thermal profile

RT	Activation Taq Polymerase	Denaturation	Annealing	Elongation	Final elongation	On hold
50°C<br>30 min	95°C<br>15 min	94°C<br>30 sec	52°C<br>30 sec	72°C<br>40 sec	72°C<br>5 min	4°C<br>∞
		45 cycles				

Product of amplification is 603 bp.
