# Supplementary material for: An inter-laboratory trial as a tool to increase rabies diagnostic capabilities of Sub-Saharan African Veterinary laboratories
Source: PLoS Negl Trop Dis. 2020 Feb 10;14(2):e0008010. doi: 10.1371/journal.pntd.0008010 (PMC7010240; doi:10.1371/journal.pntd.0008010)
Supplement: S3 Appendix — (PDF) [file pntd.0008010.s003.pdf]

Proficiency test for rabies diagnostic

(PT 2017)

Questionnaire

1- Rabies diagnostic:

1.1 Which type of diagnostic methods is applied at your laboratory?

Immunofluorescence (IF)	Virus isolation in cells	conventional RT-PCR or real-time RT-PCR	Other (precise)

2- Past experience of the laboratory:

2.1 Specify the number of diagnostic tests performed in 2016 and 2017 as well as the number of positive and negative results. (Specify the number in the empty cell. Add the number "0" if no tests were performed).

	2016	2017
**Number of dog's samples tested**		
Number of dog's positive samples for rabies		
Number of dog's negative samples for rabies		
**Number of other domesticated animals samples (other than dogs) tested**		
Number of domesticated animals positive samples		
Number of domesticated animals negative samples		
**Number of wildlife animals samples tested**		
Number of wildlife animals positive samples		
Number of wildlife animals negative samples		
**Total number of positive cases for rabies**		

2.2 Please specify the number of test performed for other countries, as well as the number of positive and negative samples, for the last two years:
………
………
………

3 – Identification of rabies virus by immunofluorescence (IF)

3.1 How is your laboratory preparing the samples?

By opening the skulls	By the occipital foramen route	By retro-orbital route

3.2 Precise which type of disinfecting solution is used to decontaminate the sampling tools. Please indicate its final concentration, for how long the tools are left to disinfect and any other supplementary information regarding the disinfecting protocol used in your laboratory.
………
………
………..

3.3 Are the slides prepared under a biological safety cabinet?

Yes	No

3.4 Which technique is used to prepare the slides?

Smear	Print	Both

3.5 Which part of the central nervous system (CNS) is used for the diagnostic?

Ammon's horn	Medulla	Cerebellum	Cortex	Spinal cord	Others

3.6 How many slides are prepared for each sample? (specify the number slides prepared for biting animals and putrefied samples).
………
………
……………………………………….

3.7 Which technique of fixation is applied?

Heat	Acetone	Heat and acetone

3.7.1 If using acetone to fix the slides, please indicate the time of incubation and the temperature of the fixation step:
………
………
………..

3.7.2 If using acetone to fix the slides, are positive and negative controls fixed in separate recipient?

Yes	No

3.7.3 How often is the acetone replaced?

Every day	Every set of samples analysed	Once a week	After each positive sample	Other

3.8 Do you use a commercial conjugate?

Yes	No

If yes, precise the name of the conjugate, the name of the brand, the working dilution used at your facilities and the expiry date?
……
……
………..

3.9 Is the blue Evans solution added to the conjugate?

Yes	No

If yes, please indicate the percentage used:
……………………….

3.10 Which solution is used for the washing step?

PBS	Water	others (specify)

3.11 The washing step is performed by dipping the slides in the solution or by rincing? Please indicate the numbers of washes:
……
……
………..

3.12 Please precise the pH of the mounting medium?
……..

3.13 Is the mounting medium made at your facilities or is it commercially bought? Please precise the name of the product, the name of the brand and the composition.
……
……
…………

3.14 By how many people is the reading of the slides performed?

1	2	>2

4 – Confirmation of rabies diagnostic using conventional RT-PCR:

4.1 Do you routinely use conventional RT-PCR to confirm the diagnostic of doubtful samples or samples from biting animals?

Yes	No

4.2 Did you use the protocol recommended by the Istituto Zooprofilaticco Delle Venezie?

Yes	No

If not, please precise which protocol was applied for the conventional RT-PCR confirmation test.
……
……

4.3 Were the samples for RT-PCR prepared at the same time as the slides for IF?

Yes	No

If no, please precise when.
……

4.4 Was the RNA extraction performed directly after sample preparation?

Yes	No

If not, please precise:

- The lapse of time between sample preparation and RNA extraction?
…………………………………………………………………………………………………….

- The temperature at which the samples were kept? ……………………………………………………………………………………………………..

4.5 Which method was used to extract RNA, if using a RNA extraction kit, please precise the name of supplier, the name of the kit and the expiry date.
……
……
……

4.6 Which final volume is used to elute the RNA?
…….

Comments :
……
……
……
……
……
……………………………………………………………………………………
