# Supplementary material for: An inter-laboratory trial as a tool to increase rabies diagnostic capabilities of Sub-Saharan African Veterinary laboratories
Source: PLoS Negl Trop Dis. 2020 Feb 10;14(2):e0008010. doi: 10.1371/journal.pntd.0008010 (PMC7010240; doi:10.1371/journal.pntd.0008010)
Supplement: S1 Table — The concordance, specificity, sensitivity and K coefficient with p-value were calculated omitting sample S10. Compared to the results analysis including all ten PT samples, the concordance, the specificity and the kappa, respectively increased from 87.7% to 90.6%, 86.2% to 92.3% and 0.59 to 0.66. No statistically significant differences between the concordance, the specificity and the kappa including or excluding sample S10 were established. *P < 0.05; ** P < 0.01; *** P < 0.001; ns = not significant. (PDF) [file pntd.0008010.s004.pdf]

| Laboratory | True positive | True negative | False positive | False negative | Concordance (%) | Sensitivity (%) | Specificity (%) | Kappa |
|---|---|---|---|---|---|---|---|---|
| L01 | 5 | 4 | 0 | 0 | 100 | 100 | 100 | 1** |
| L02 | 5 | 4 | 0 | 0 | 100 | 100 | 100 | 1** |
| L04 | 5 | 4 | 0 | 0 | 100 | 100 | 100 | 1** |
| L05 | 5 | 4 | 0 | 0 | 100 | 100 | 100 | 1** |
| L06 | 3 | 4 | 0 | 2 | 77.8 | 60 | 100 | 0.57* |
| L07 | 5 | 4 | 0 | 0 | 100 | 100 | 100 | 1** |
| L08 | 5 | 4 | 0 | 0 | 100 | 100 | 100 | 1** |
| L09 | 5 | 3 | 1 | 0 | 88.9 | 100 | 75 | 0.77** |
| L10 | 5 | 4 | 0 | 0 | 100 | 100 | 100 | 1** |
| L11 | 3 | 4 | 0 | 2 | 77.8 | 60 | 100 | 0.57* |
| L12 | 4 | 4 | 0 | 1 | 88.9 | 80 | 100 | 0.78** |
| L13 | 4 | 3 | 1 | 1 | 77.8 | 80 | 75 | 0.55* |
| L14 | 4 | 2 | 2 | 1 | 66.7 | 80 | 50 | 0.3 (ns) |
| **Overall** | **58** | **48** | **4** | **7** | **90.6** | **89.2** | **92.3** | **0.66***** |
